# Anti-HIV, antitumor and immunomodulatory activities of paclitaxel from fermentation broth using molecular imprinting technique

**DOI:** 10.1186/s13568-019-0915-1

**Published:** 2019-12-03

**Authors:** Junhyok Ryang, Yan Yan, Yangyang Song, Fang Liu, Tzi Bun Ng

**Affiliations:** 1 Department of Microbiology, The Key Laboratory of Molecular Microbiology and Technology, Ministry of Education, College of Life Science, Nankai University, Tianjin, 300071 China; 2 School of Biomedical Sciences, Faculty of Medicine, The Chinese University of Hong Kong, Shatin, New Territories Hong Kong, China

**Keywords:** Paclitaxel, Taxol, Antitumor activity, Anti-HIV activity, Molecularly imprinted polymer, Immunomodulatory activity

## Abstract

In this study, a single component paclitaxel was obtained from fermentation broth by molecular imprinting technique, and its antiviral, antitumor and immunomodulatory activities were studied. The results showed that paclitaxel had a good inhibitory activity on human breast cancer MCF-7 cells and showed a concentration- dependent relationship with an IC50 of about 15 μg/mL in the sulforhodamine B assay. At the same time, paclitaxel exerted a weak inhibitory activity on cervical cancer Hela cells. In addition, paclitaxel not only inhibited the invasion of HIV-1 pseudovirus into cells, but also exhibited inhibitory activity to a certain extent after viral invasion of the cells. At a paclitaxel concentration of 20 μg/mL, the inhibition of HIV-1 pseudovirus reached about 66%. The inhibition of HIV-1 protease activity was concentration-dependent. At a concentration of 20 μg/mL, the inhibitory effect of paclitaxel on HIV-1 protease was similar to that of the positive control pepstatin A, being 15.8%. The HIV-1 integrase inhibiting activity of paclitaxel was relatively weak. Paclitaxel significantly up-regulated the expression of interleukin-6.

## Introduction

Among plant-derived natural products, paclitaxel (C_47_H_51_NO_14_), which was first isolated from the bark of the Pacific yew *Taxus brevifolia*, also known commercially as taxol, is a chemotherapeutic diterpenoid drug that exhibits potent anticancer activity (Kasaei et al. 2017; Zhou et al. 2010).

Due to its complex structure, unique medicinal mechanism and good anti-tumor activity, paclitaxel has been the subject of research of many scholars (Lasala et al. 2006; Oberlies and Kroll 2004). Moreover, paclitaxel has been studied for its potential for treating other diseases including neurodegenerative diseases and polycystic kidney disease (Zhang et al. 2005) and for the prevention of restenosis (Herdeg et al. 2000). Thus, paclitaxel is in high demand, and research interest in paclitaxel will continue to escalate (Li et al. 2017).

At present, the main source of paclitaxel is still dependent on the yew tree. Due to the increasing demand for paclitaxel, yew tree is on the verge of extinction, hence it is urgent to find a new way to produce paclitaxel (Ismaiel et al. 2017). Thus researchers aim at taxol production by means of several modern techniques including chemical synthesis, semi-synthesis method and plant tissue culture method. Nevertheless, these methods have both advantages and disadvantages (Ismaiel et al. 2017; Shankar Naik 2019). Kusari et al. (2012) reported that there has been tremendous interest in locating alternative sources of paclitaxel, including fungal endophytes (Kusari et al. 2012). In general, microbial fermentation has demonstrated that isolation and identification of taxol-producing fungi is a good strategy in the production of taxol (Ismaiel et al. 2017). Thus, developing a cost-effective paclitaxel fermentation process by microorganisms has become a sustainable solution (Shankar Naik 2019; Somjaipeng et al. 2015).

Molecular imprinting technology is a technique for preparing a polymeric material with the ability for recognizing a specific target molecule (template molecule). With the advancement of science and technology, molecular imprinting is now a mature technology, and numerous researchers deploy this technology to separate and obtain the target product (Li et al. 2017).

Therefore, MIP is widely employed for the separation and enrichment of active ingredients of natural products (Ishkuh et al. 2014). In recent years, the application of MIP technology in the separation of active ingredients of natural medicinal resources has received more and more attention.

For instance, some researchers have extensively investigated the interactions between paclitaxel and some common functional monomers, such as methacrylic acid (MAA), acrylamide (AM), 2-vinylpyridine (2-VP), and 4-vinylpyridine, in different solvents by ultraviolet spectrophotometry and found that the strongest interaction between paclitaxel and 2-VP in chloroform was observed at a ratio of 1:6 (Li et al. 2013, 2015). Ishkuh et al. (2014) have employed MSP using ethylene glycol dimethacrylate for preparing MIPs for paclitaxel with a high degree of crosslinking and found that the highest binding capacity for paclitaxel was 48.4%. However, the particle sizes of the MIPs were mainly distributed around 100 nm. Hence, despite its excellent imprinting effect, the MIPs could not be used further for separation and analysis (Ishkuh et al. 2014).

Compared with conventional separation techniques such as liquid–liquid extraction and column chromatography, molecular imprinting technology has the advantages of economy, speed and simplicity (Li et al. 2017).

At present, a large number of studies have established that paclitaxel exhibits high anticancer activity. Its anti-tumor mechanism involves binding to tubulin, formation of a stable tube bundle, leading to the loss of balance between dimers, and promotion of microtubule assembly polymerization (Li et al. 2017). Consequently, the cancer cells are arrested in the late G or M phase, mitosis of the cancer cells is inhibited, proliferation of the cancer cells is impeded, and the cells gradually shrink and eventually die. However, there are very few reports on other biological activities of paclitaxel such as inhibition of HIV-1 viral replication activity and regulation of immunity (Shankar Naik 2019; Wang et al. 2015). Wang et al. (2015) compared activities of taxol produced by endophytic fungi *Nodulisporium sylviforme* HDFS4-26 with that of taxol extracted from yew bark in inhibiting growth and inducing apoptosis of cancer cells (Wang et al. 2015). Cellular morphology, cell counting kit (CCK-8) assay, staining (HO33258/PI and Giemsa), DNA agarose gel electrophoresis, and flow cytometry (FCM) analyses were used to determine the apoptosis status of cancer cell lines such as MCF-7 cells, HeLa cells, and ovarian cancer HO8910 cells. The fungal taxol exhibited cytotoxic activity against HeLa cancer cell lines in vitro and displayed antifungal and antibacterial activities against different pathogenic strains (Das et al. 2017).

In this study, paclitaxel samples obtained from endophytic fungus fermentation broth by molecular imprinting and solid phase extraction were used to investigate the antiviral, antitumor and immunomodulatory activities of the paclitaxel, which enriched the application value of paclitaxel. It is speculated that the intrinsic relationship between malignant tumor and AIDS is pointed out. It supports a theoretical foundation for the future diagnosis of potential diseases.

## Materials and methods

### Materials

Fermentation broth is commercial lyophilized powder (Professor Xudong Zhu Laboratory, State Key Program of Microbiology and Department of Microbiology, College of Life Sciences, Nankai University, Tianjin, China). 4-Vinylpyridine (4-vp) was purchased from Across Organics (USA). Methacrylic acid (MAA) and ethylene glycol dimethacrylate (EGDMA) were obtained from Aldrich (USA). Acrylamide (AA) was obtained from Union Star Biotechnology Co., Ltd, Tianjin, China. 2,2′-azobisisobutyronitrile (AIBN) (Kuwait Company, Tianjin, China) was recrystallized in ethanol before use. Dimethyl sulfoxide (DMSO) was purchased from Sigma (USA). Paclitaxel (> 98%) was purchased from Shanghai Jinhe Biotechnology Co., Ltd. Methanol, acetone, tetrahydrofuran and isooctane were of HPLC grade. Other reagents were of analytical grade.

### Cell lines and cell culture

All cell lines were kindly provided by Professor Wentao Qiao (Department of Microbiology, Nankai University) and maintained in DMEM supplemented with 10% fetal bovine serum (Gibco, Invitrogen), 100 IU/mL of penicillin and 100 μg/mL of streptomycin at 37 °C in a humidified atmosphere of 95% air/5% CO_2_.

### Preparation of molecularly imprinted polymers

MAA was selected as the functional monomer. Acetone was the porogen, EGDMA was the crosslinking agent, AIBN was the thermal initiator, and the ratio of the template: functional monomer: crosslinking agent used was 1:6:30. The template was synthesized. Affinity and transfer selectivity of molecularly imprinted polymers. The template molecules were removed when preparing the non-imprinted polymer, and the remaining steps were performed in the same manner as described above for the imprinted polymer.

### MIP-SEP procedures

Two hundred milligrams of the prepared polymer as a filler were accurately weighed, and added to an empty solid phase extraction column (3 mL, 8 mm in diameter). The solid phase extraction column was made of polypropylene. The commercial joints and interfaces have been standardized and can be directly connected to the vacuum device. The upper column sample was dissolved in methanol: water (2:8, v/v), while the SPE cartridge was equilibrated with the same mixture of methanol and water. A mixed solution of methanol and water was used as a washing solution in the solid phase extraction process. Using methanol: glacial acetic acid (9:1, v/v) as eluent, the collected eluent was rotary evaporated to remove all solvents, and then the enriched product was dissolved in 2 mL of methanol, and analyzed by HPLC.

### Preparation of samples

Ten gram lyophilized fermentation broth was dissolved in 100 mL of distilled water and filtered. The filtered liquor was evaporated under reduced pressure and then dissolved in 100 mL of methanol.

The extract was partitioned in a mixture of dichloromethane: *n*-hexane: methanol (5:4:1, v/v/v) and the fraction in the lower layer was evaporated under reduced pressure. The crude paclitaxel was dissolved in a mixture of methanol: water (1:9, v/v) and processed as described in 2.3.

### High-performance liquid chromatographic (HPLC) analysis

The analysis was performed using a LabAlliance high-performance liquid chromatographic instrument equipped with an ultraviolet detector at 280 nm. All separations were achieved on an analytical reversed-phase Kromasil 100-5 C18 column ($4.6\;{\text{mm}} \times 250\;{\text{mm}}$). The injection volume was 20 μL, and the flow rate was maintained at 1.0 mL/min. H_2_O 0.05% HAc MeOH (30:70) was used as a mobile phase.

### Assay of antitumor activity

The inhibitory effects on tumor cell lines were determined using the protein-staining sulforhodamine B (SRB) assay based on the ability of the SRB dye to bind basic amino acid residues on proteins (Skehan et al. 1990) which is similar in performance to the MTT (dimethylthiazol-diphenyltetrazolium bromide) assay as assays of cytotoxicity. Different human cell lines, including embryonic kidney 293T, cervical cancer HeLa and breast cancer MCF-7 cells were taken as targets for the paclitaxel sample. The various cell lines were maintained in DMEM medium supplemented with streptomycin, penicillin and FBS at 37 °C in a humidified atmosphere of 5% CO_2_. 100 μL, cell suspension (1 × 10^5^ cells/mL), with cells in the exponential growth phase, were seeded into each well of a 96-well culture microplate. After incubation for 24 h, paclitaxel solution was added and incubation was continued for another 48 h. The cells were fixed in cold trichloroacetic acid (25 μL, 50%) and stained with 0.4% SRB solution. The protein-bound SRB dye was solubilized with 100 μL Tris–HCl buffer (10 mM, pH 7.4) for determination of the optical density (OD) at 490 nm. The negative control was composed of cells treated with DMSO. The vehicle control was composed of cells without any treatment.

$$Cell\;viability \,\left( \% \right) = \left( { mean\;OD\;of\;treated\;cells/mean\;OD\;of\;vehicle\;treated\;cells} \right) \times 100\%$$

$$Inhibition\;Rate \,\left( \% \right) = \left( {1 - mean\;OD\;of\;treated\;cells/mean\;OD\;of\;vehicle\;treated\;cells} \right) \times 100\% .$$

### Assays of anti-HIV activity

#### Assay of inhibition of HIV-1 entry

TZM-BL-croGFP cells were cultured at 37 °C, in 5% CO_2_. When the cell density was 80–90%, the cells were digested with trypsin, and the cells were collected at 1000*g* for 3 min. The PBS buffer was washed repeatedly 3 times. The cell density was adjusted to 10^5^ cell/mL. The MIPs of different concentrations to be tested were added 2 h before the addition of HIV-1 pseudovirus and 2 h after the addition, and the culture was continued for 24 h. The medium in the well was aspirated and 150 μL of cell lysate was added. The cells were fully lysed by reaction at room temperature for 15 min.

After the lysis was completed, the cell lysate was collected and the supernatant was collected by centrifugation at 5000*g* for 1 min. Then 200 μL of the supernatant were removed and mixed evenly with 50 μL of luciferase substrate solution in a 96-well plate. The mixture was placed in a luciferase luminescence detector for determination of the fluorescence intensity at 560 nm (Is). The blank control was free of pseudovirus (*Ib*). The negative control consisted of only the pseudovirus but without the sample (*In*). AZT was used as the positive control.

The inhibition of the entry of pseudovirus into the cell was calculated as follows:

$$Inhibition\;rate \,\left( \% \right) = \left( {In - Is} \right) / \left( {In - Ib} \right) \times 100\% .$$

#### Assay of HIV-1 protease inhibitory activity

The strain used was *E. coli* BL21-pPR (a plasmid containing the HIV-1 protease gene). The cells were cultured in LB medium for 12 h. Then 50 μL of the bacterial solution was transferred to 4 mL of fresh LB liquid medium containing 50 μg/mL kanamycin sulfate. The test sample and 40 μM inducer IPTG were added before culture at 37° C.

A 100 μL aliquot was taken at hourly intervals into a 96-well plate. The growth of the cells was determined by measurement of absorbance at a wavelength of 490 nm. The cell culture time was taken as the horizontal and vertical coordinates, and the absorbance was plotted on the ordinate. The growth curve of the cells was drawn, the slope K was calculated, and the inhibition rate of the HIV-1 protease expression was calculated according to the slope. The protease inhibitor Pepstatin A was used as a positive control; in the negative control, LB medium was used instead of the sample, and the absorbance was K0; in the blank control, the LB medium was used instead of the sample, and the absorbance was K1, but no IPTG was induced; the absorbance of each sample system was Ks.

The HIV-1 protease inhibitory activity was calculated as follows:

$$Inhibition\;rate \,\left( \% \right) = \left( {Ks - K0} \right) / \left( {K1 - K0} \right) \times 100\% .$$

#### Assay of HIV-1 integrase inhibitory activity

The plasmid pET28a-LTR cloned with HIV-1 LTR was transformed into *E. coli* DH5(R) strain, and the plasmid was extracted after expansion and culture. Then the following 10-μL reaction system was constructed: 1 μL Tris–HCl (20 mM, pH 8.0) buffer, 1 μL β-mercaptoethanol (2 mM), 1 μL MnCl2 (2 mM), 1 μL sample at different concentrations, 3 μL substrate plasmid and 3 μL of HIV-1 integrase (10 pmol) were incubated at 37 °C for 30 min followed by agarose gel electrophoresis.

In this experiment, the control was DBZ, the negative control was Tris–HCl buffer (10 mM, pH 7.4) instead of the sample, and the blank control was Tris–HCl buffer instead of the sample and no HIV-1 integrase was added.

#### Determination of cytokine gene expression levels

Female BALB/c mice (7 weeks old, 20–25 g) were obtained from the Chinese Academy of Military Medical Sciences Laboratory Animal Center. The procedures of all animal experiments had been approved by the Chinese Academy of Military Medical Sciences Animal Research Ethics Committee. Ten BALB/c mice were randomly divided into 2 groups, a control group and a paclitaxel group, with 5 mice in each group. Normal saline was injected intraperitoneally into the control group. The MIPs group was injected intraperitoneally with a dose of 50 mg/kg for 7 consecutive days and once daily. On the 8th day, both groups of mice were treated with lipopolysaccharide at a dose of 3 mg/kg and euthanized after 12 h.

The total splenic RNA was isolated by using the Trizol kit. The reaction mixture was composed of 1–5 μg total RNA, 0.5 mM dNTPs, 50 ng oligo (dT) primer, reverse transcription buffer, and 3 units of reverse transcriptase. It was heated at 65 °C for 5 min, then conducted at 42 °C for 30 min, and finally inactivated by heating to 95 °C for 5 min. The primers for real-time quantitative PCR were shown in Additional file 1: Table S1 and the system for real-time quantitative PCR was shown in Additional file 1: Table S2.

The following thermocycler program was used for real-time PCR: 1 min pre-incubation at 95 °C, followed by 40 cycles of incubation at 94 °C for 15 s, 55 °C for 30 s, 72 °C for 45 s. The 2^−ΔΔCt^ method was used to analyze the results. GAPDH was the internal control.

Determination of cytokine gene expression levels was calculated as follows:

$$\Delta {\text{Ct}} = Ct_{{\left( {Target\;gene} \right)}} - Ct_{GAPDH} \;\left( {{\text{amplification}}\;{\text{from}}\;{\text{the}}\;{\text{same}}\;{\text{cDNA}}} \right)$$

$$\Delta \Delta {\text{Ct}} = \Delta Ct_{{\left( {Treated} \right)}} - \Delta Ct_{{\left( {Control} \right)}} .$$

### Statistical analyses

Results were expressed as mean ± standard deviation (SD). Statistical significance was evaluated using analysis of variance (ANOVA, SPSS software version 22; IBM Corp., NY) test followed by the least significant difference (LSD) test at *p* ≤ 0.05 level.

## Results

### Preparation and HPLC analysis of paclitaxel by molecularly imprinted method

**Fig. 1:**
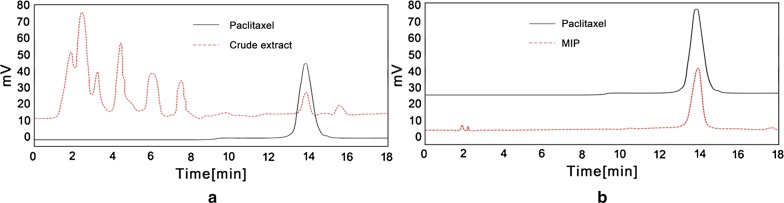
HPLC diagram of paclitaxel from the fermentation broth (**a** paclitaxel standard and crude extract, **b** paclitaxel standard and MIPs)

The presence of paclitaxel in the crude extracts was confirmed by HPLC analysis. As shown in Fig. 1a, in the fermentation broth, compared with paclitaxel standard, starting peak appeared at the same retention time of about 13 min, which proves that the crude extract from the fermentation broth contains paclitaxel, by the way, the composition of the product was complex as well as the content of paclitaxel was smaller than that of other ingredients.

Figure 1b shows that, after crude extract was treated by the molecularly imprinted column, the impurities were almost completely separated, and paclitaxel in the sample was considerably enriched and the homogeneity was greatly enhanced.

Compared with the paclitaxel standard, the target material was seldom contained in the crude extract. The solution of MIPs eluted was mostly paclitaxel with only a minute extraneous peak. It proved that the synthesized molecularly imprinted polymer had a substantial enriching effect on the extraction of paclitaxel from the fermentation broth.

### Morphological characterization of the MIPs

**Fig. 2:**
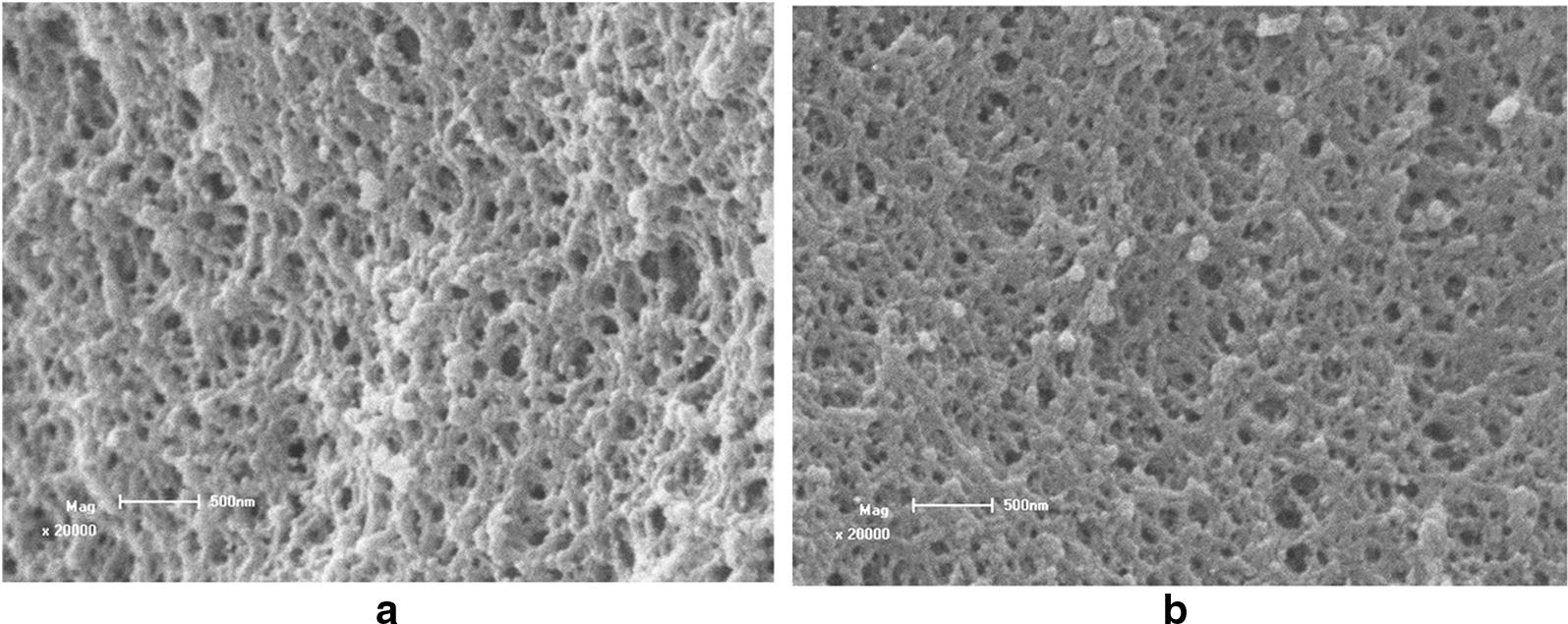
Scanning electron micrographs of the MIPs (**a**) and NIPs (**b**)

The apparent morphology of the polymer surface was observed directly by SEM to gain an intuitive understanding of the polymer. In the MIPs, the roughness of the particle surface itself causes the increase in the surface area compared with the NIPs, which possessed a uniform, compact, and smooth shape. The nonporous structure in the NIPs particles was due to the lack of specific binding sites which were created for MIPs and suggested that the MIPs had great potential in application as sorbents (Fig. 2).

### Effect of paclitaxel on tumor cell proliferation

**Fig. 3:**
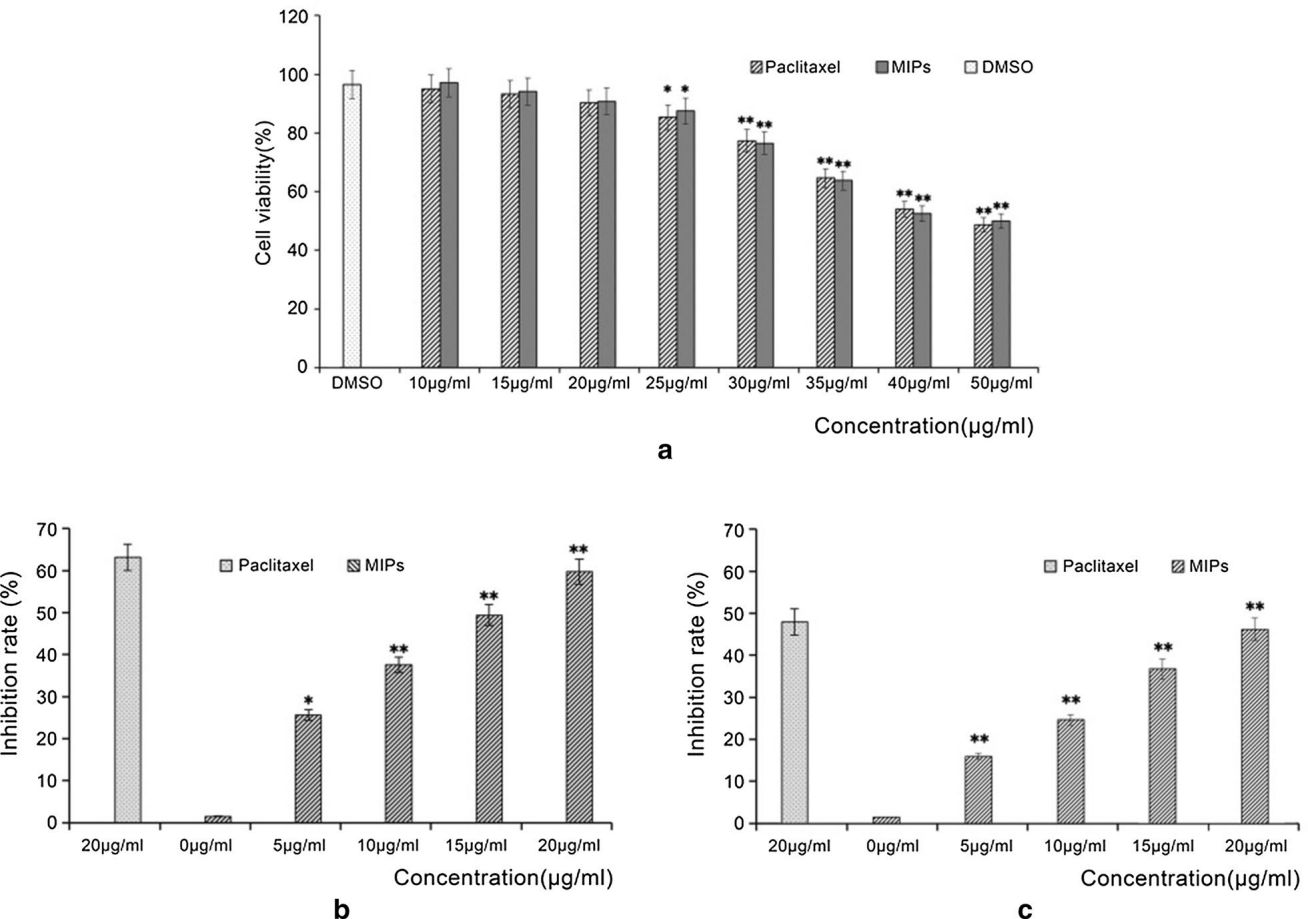
Antitumor activity of paclitaxel. **a** Cytotoxicity of paclitaxel on 293T cells; **b** inhibitory activity of paclitaxel on MCF-7 cells; **c** inhibitory activity of paclitaxel on Hela cells. *p < 0.05, **p < 0.01 versus non-treatment with MIPs

As shown in Fig. 3a, when MIPs concentration was 20 μg/mL, there was no significant effect on embryonic kidney 293T cells (*p* > 0.05). Moreover, the damage to the cells caused by the addition of DMSO was negligible.

As can be seen from Fig. 3b, the antitumor activity of the paclitaxel standard was slightly higher than that of MIPs at the same concentration. However, MIPs also exhibited good antitumor activity with an IC_50_ of 15 μg/mL for human breast cancer MCF-7 cells in a concentration- dependent manner. In Fig. 3c, paclitaxel inhibited cervical cancer Hela cells by 40% at 20 μg/mL, and the antitumor activity against Hela cells was significantly lower than that of MCF-7 cells, which might be related to the mechanism of action. However, concentration dependence was also observed.

### Inhibitory effect of paclitaxel on the entry of HIV-1 pseudovirus

The life cycle of hiv shows HIV-1 enters host cells after 1–2 h of infection, adsorption inhibitors must be added before HIV-1 infects host cells to have a corresponding effect. In this study, samples were added before and 2 h after infection. AZT (zidovudine, a listed nucleoside reverse transcriptase inhibitor) was used as the control group to test the inhibitory effect of the paclitaxel of different concentrations on the entry of hiv-1 pseudovirus into TZM-BL cells.

**Fig. 4:**
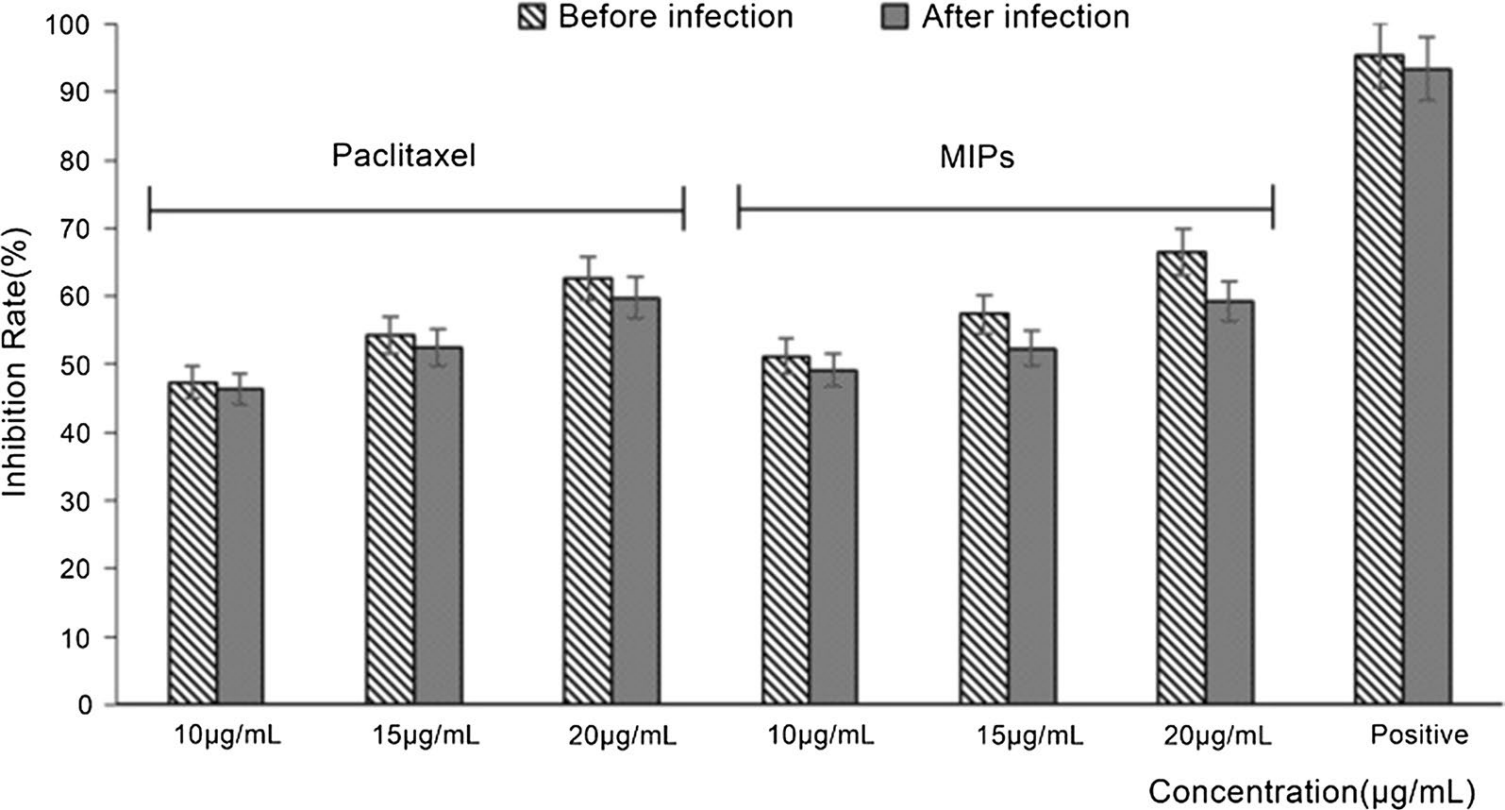
Inhibitory effects of paclitaxel on HIV-1 entrance activation induced by pseudovirus. “Positive” refers to the positive control AZT

As shown in Fig. 4, the positive control azidothymidine (AZT) maintained potent inhibitory activity before and 15 h after infection. The inhibition rate was 90%, and there was no significant difference before and after infection ($p > 0.05$). This indicates that AZT can inhibit the entry of HIV-1 pseudovirus to a certain extent, but it does not target the process of virus invasion.

In the pre-protected group, both the paclitaxel standard and MIPs manifested good inhibitory activity, although the paclitaxel sample displayed better and concentration-dependent inhibitory activity. Although the inhibition rate decreased after 2 h of infection, the difference was not significant ($p > 0.05$), indicating that the paclitaxel sample had similar inhibitory activity toward AZT in the process of HIV-1 pseudovirus entry. The target is not limited to viral entry.

### Inhibitory effect of paclitaxel on HIV-1 protease activity

**Fig. 5:**
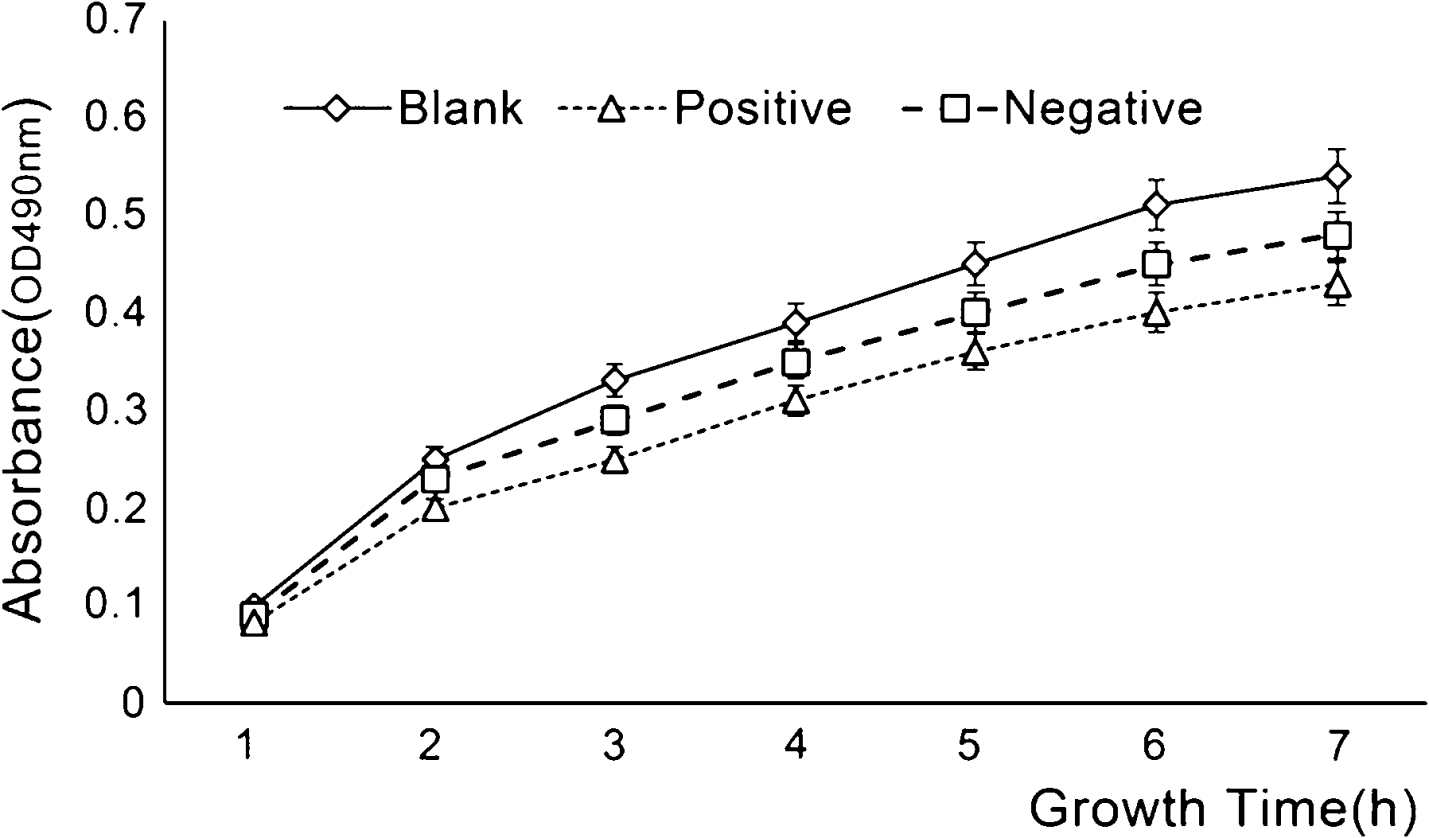
Effect of HIV-1 protease on IPTG-induced *E. coli* growth. (Positive control: *E. coli* in the presence of IPTG and pepstatin A; negative control: *E. coli* in the presence of IPTG; blank control: *E. coli* only)

Pepstatin A (an HIV-1 protease inhibitor) served as a positive control. At 80 μg/mL, pepstatin A brought about a certain degree of recovery of cell growth (Fig. 5). As shown in Fig. 5, the paclitaxel sample inhibited HIV-1 protease, but the naturally extracted paclitaxel sample inhibited protease activity to a less extent than the paclitaxel standard. This might be due to the damage of the biological activity of paclitaxel caused by the extraction process. However, at an effective concentration of 20 μg/mL, the inhibitory effect of paclitaxel on HIV-1 protease was approximately similar to that of the positive control pepstatin A (80 μg/mL). Thus paclitaxel demonstrated good HIV-1 protease inhibitory activity.

**Fig. 6:**
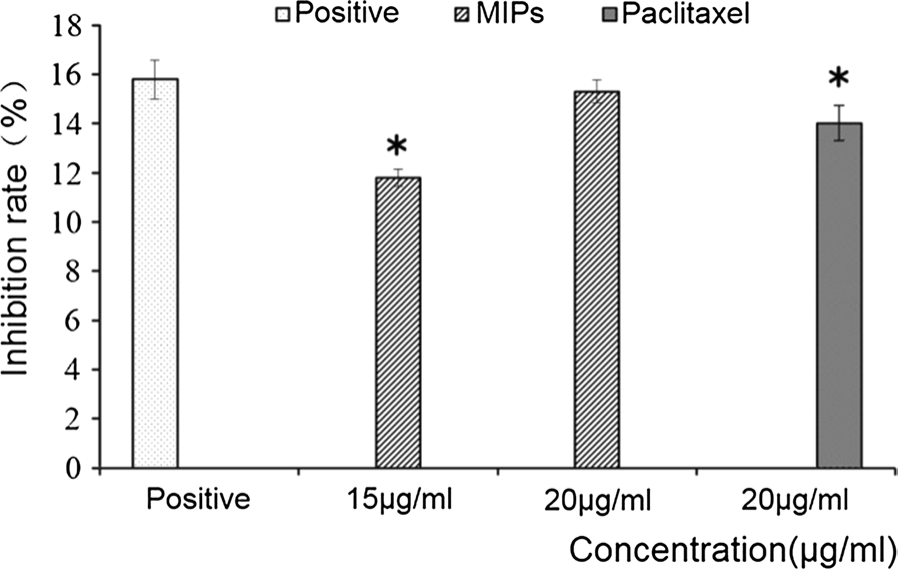
Inhibitory effects of different concentrations of paclitaxel, MIPs and pepstatin A on HIV-1 protease. *p < 0.05, **p < 0.01 versus positive

### Inhibitory effect of paclitaxel on HIV-1 integrase activity

**Fig. 7:**
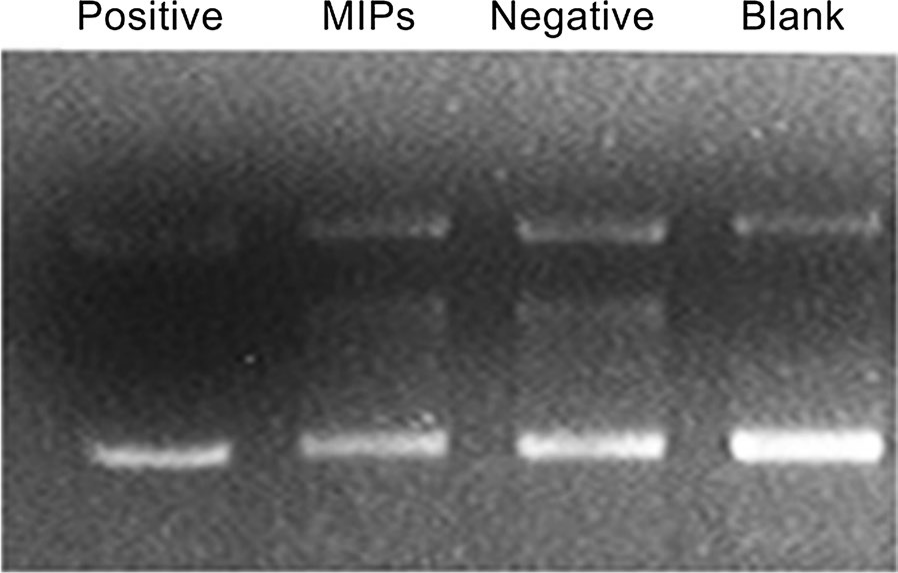
Inhibitory effect of paclitaxel on HIV-1 integrase

In the in vitro assay model, the purified His-tagged HIV-1 integrase was applied to the substrate plasmid pET28a-LTR using HIV-1 integrase. The cleavage activity of the sample inhibiting HIV-1 integrase was characterized by detecting changes in plasmid linearity. The positive control used was raltegravir (the only commercially available HIV-1 integrase inhibitor). As shown in Fig. 7, raltegravir was able to significantly inhibit integrase cleavage activity, and the open-loop state of the plasmid under its action was comparable to that of the blank control group. The inhibitory activity of MIPs was better than the negative control.

### Effect of MIPs on cytokine gene expression

**Fig. 8:**
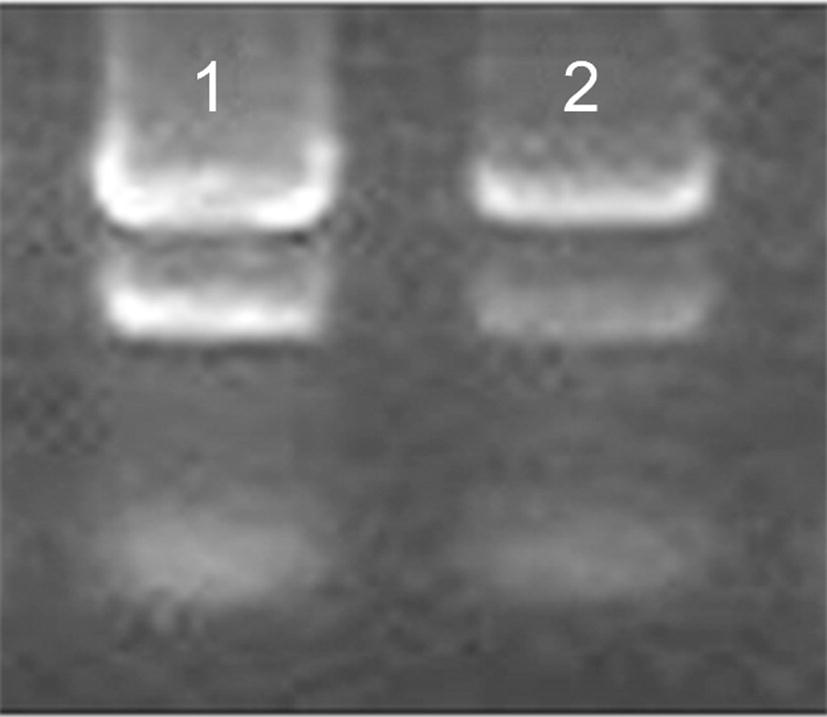
Total RNA in mouse spleen lymphocytes. 1: 0.9% NaCl, 2: MIPs (paclitaxel)

As shown in Fig. 8, the total RNA of mouse spleen lymphocytes was clearly separated into three bands. The upper two bands were 28s rRNA and 18s rRNA. The two bands were well separated and the integrity of the sample was good, and the next one was 5s rRNA. The A260/A280 values were 2.05 and 2.05 respectively, indicating high RNA purity, and the samples could be reverse transcribed and further experiments could be conducted.

**Fig. 9:**
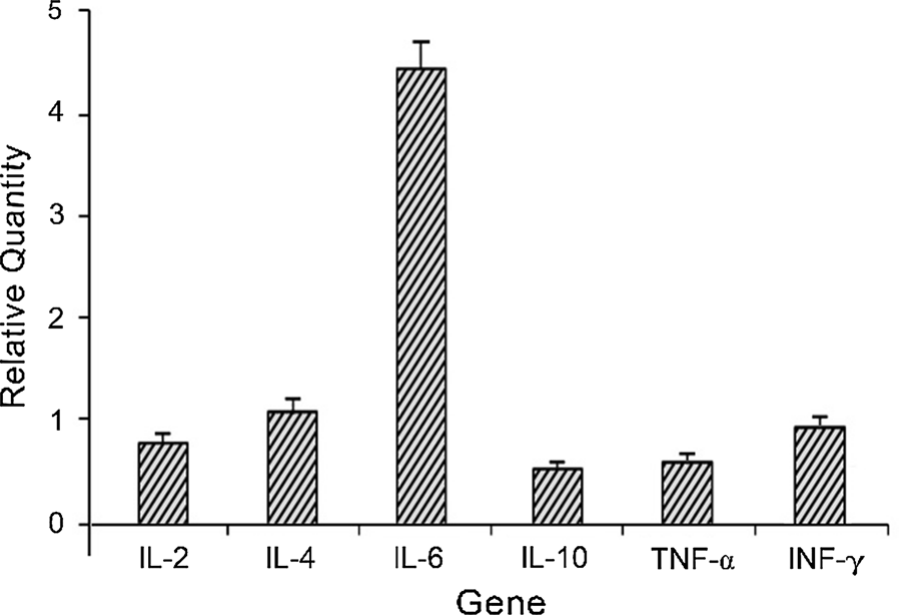
Effects of MIPs on the expression of target genes (n = 5)

The increase of DNA products was monitored in real time by determining the change of fluorescence intensity. As shown in Fig. 9, after the intraperitoneal injection of MIPs, there were some changes in the cytokines, especially the expression of IL-6 was significantly up-regulated. IL-6 responded to tissue damage and stimulated the production of other cytokines. As an anti-inflammatory factor, IL-6 can inhibit TNF-α, IL-1 and IL-10. It was found from the table that both TNF-α and IL-10 were down-regulated, while IFN-γ and IL-4 remained almost unaltered.

## Discussion

Paclitaxel (taxol) belonging to a class of complex diterpenoids, called taxanes is a effective anti-cancer drug against breast cancer, and it has received extensive attention due to its unique anticancer mechanism. Initially, paclitaxel was isolated from the bark of yew (Shankar Naik 2019). Since then, paclitaxel has been isolated from some plants belonging to genus *Taxus* (family *Taxaceae*, syn Coniferales) and other genera of the same family such as *AmenoTaxus*, *Austro Taxus*, *PseudoTaxus* (Flores-Bustamante et al. 2010; Hao et al. 2013). The traditional methods of extracting paclitaxel from the bark of *Taxus* species have the disadvantage of high cost and environmental damage. Unprecedented yew cutting, low amounts of paclitaxel production, laborious and slow process of paclitaxel extraction prompted the discovery of the alternative source of paclitaxel (Flores-Bustamante et al. 2010). Thus researchers focus on paclitaxel production by means of several modern techniques, for example chemical synthesis, and plant tissue culture (Jennewein and Croteau 2001) and microbial fermentation etc. (Frense 2007; Visalakchi and Muthumary 2010). Each method has its own pros and cons. However, by microbial fermentation method, it is easy to reduce costs of production and increase the yield of paclitaxel, which is very economic and practical. In general, microbial fermentation has demonstrated that isolation and identification of taxol-producing fungi is a good strategy in the production of paclitaxel.

Molecular imprinting technology is now mature, and many researchers use this technology to separate and obtain the desired product (Li et al. 2013, 2015). In the process of synthesizing molecularly imprinted polymers, the difficulty lies in the choice of functional monomers and porogens. Since the structure of paclitaxel has various functional groups such as a phenolic hydroxyl group, an ester group, an amino group, and a hydroxyl group, it has both an acidic as well as a basic functional monomer (Li et al. 2015).

Previously, we studied MAA (acidic), AM (neutral) and 4-vp (alkaline) as functional monomers. The results showed that when MAA was used as a functional monomer, it had good specificity for the enrichment of paclitaxel, and the recovery attained was 70%. At the same time, acetone was found to have good specificity as a porogen, and the recovery was 83%. In addition, when the ratio of the template: functional monomer: cross-linker was 1:6:30, the adsorption rate and recovery rate of the paclitaxel sample are the highest.

After the molecularly imprinted polymer was determined, the crude paclitaxel in the bark of the yew was first examined by HPLC to determine whether it had an enrichment effect (Li et al. 2017). Figure 1 shows that a single component consistent with the retention time of the paclitaxel standard was obtained, indicating that the synthesized molecularly imprinted polymer has a specific enrichment effect on the paclitaxel sample.

Paclitaxel has been reported to have good antitumor activity, especially against MCF-7 cells (Kasaei et al. 2017; Wang et al. 2015). In this study, we examined the antitumor activity of the enriched paclitaxel samples. The experimental results show that the paclitaxel sample enriched by the synthesized molecularly imprinted polymer exhibited a good anti-proliferative activity toward both MCF-7 and Hela cells (Figs. 3, 4). Moreover, the antitumor activity of the paclitaxel sample was higher than that of the paclitaxel standard. It is speculated that the activity of the natural product was retained, and the antitumor activity was displayed.

Up to now, there are few reports on the antiviral activity of paclitaxel (Krawczyk et al. 2005; Stebbing et al. 2003), in particular, there are very few reports on anti-HIV activity of microbial paclitaxel by molecular imprinted polymer. In this paper, we explored the potential of paclitaxel in the prevention and treatment of AIDS and conducted research in three areas: HIV-1 viral invasion, HIV-1 protease activity, and HIV-1 integrase activity. Based on the life cycle of viral replication, we designed to add paclitaxel samples 2 h before virus invading cells and 2 h after invasion, and to determine whether paclitaxel samples inhibit HIV-1 virus invasion into host cells with a fluorescence microplate reader.

Paclitaxel sample inhibited the entry of HIV-1 virus into TZM-BL cells, and addition of the pre-invasive drug had a higher inhibition rate on the virus, indicating that in future practice, the method of preventive dosing can be employed to prevent and treat AIDS more effectively (Fig. 4).

Interestingly, the paclitaxel sample not only acts on the process before the virus invades, but also has an inhibitory effect upon viral invasion of the host cells. This result provides a theoretical basis for the subsequent inhibition of HIV-1 protease and integrase activity by paclitaxel samples (Figs. 5, 6, 7).

The results showed that the paclitaxel sample had different inhibitory activities against the two enzymes and had stronger HIV-1 protease inhibitory activity. We assumed that it may be related to the model of detection. The test of integrase inhibition is an in vivo detection model that can directly act on host cells; the assay of protease inhibition is an in vitro detection model, but only the degree of cleavage of the plasmid is detected.

The body is in an immune-regulated state under normal conditions, and once stimulated by the outside world, the immune system will be disordered (Yuan et al. 2010). Therefore, we can detect the effect of paclitaxel samples from the aspect of cytokine changes. Under normal conditions, the body’s immune cells Th1/Th2 are in dynamic equilibrium (Yuan et al. 2010).

Many studies have disclosed that in the early stage of HIV-1 virus-infected host, the immune balance is disrupted, causing the immune cells Th1 to shift to Th2, and the immune-related cytokines are characterized by down-regulation of IL-2 and up-regulation of IL-4 and IL-10, and The pro-inflammatory factor TNF-α, which is closely related to the virus, is activated, allowing NF-kB to bind to the LTR of HIV-1 virus, thereby activating viral replication, causing immune imbalance and disease progression (Coghill et al. 2017; Otiti-Sengeri et al. 2018). As shown in Fig. 9, MIPs can down-regulate the expression of IL-10 and up-regulate the up-regulation of IL-6, which has a certain positive effect on balancing the cytokines in immune imbalance.

In this study, we obtained paclitaxel from the fermentation broth of endophytic fungus by molecular imprinting technology and MIPs were used to investigate the antiviral activity, antitumor activity and immunomodulatory effects of the paclitaxel. The findings enriched the application value of paclitaxel, and provided theoretical support for the development of small molecule natural products.

## Supplementary information

**Additional file 1: Table S1.** Primers for real-time quantitative PCR. **Table S2.** Systems for real-time quantitative PCR.

## Data Availability

All datasets on which the conclusions of the manuscript rely are presented in the main paper

## Abbreviations

MIPs: molecular imprinted polymers
NIP: non molecularly imprinted polymer
4-vp: 4-vinylpyridine
MAA: methacrylic acid
AA: acrylamide
EGDMA: dimethacrylate
AIBN: 2,2′-azobisiso-butyronitrile
DMSO: dimethyl sulfoxide
DMEM: Dulbecco’s modified eagle medium
MTT: dimethylthiazol diphenyltetrazolium bromide
SRB: sulforhodamine
FBS: fetal bovine serum
293T cell: embryonic kidney cell
HeLa cell: cervical cancer cell
MCF-7: breast cancer cells
HIV-1: human immunodeficiency virus-1
IPTG: isopropyl-beta-d-thiogalacto-pyranoside
GAPDH: glyceraldehyde-3-phosphate dehydrogenase
IL-2, -4, -6, -10: interleukin-2, -4, -6, -10
TNF-α: tumor necrosis factor-α
