# Supplementary material for: Anti-HIV, antitumor and immunomodulatory activities of paclitaxel from fermentation broth using molecular imprinting technique
Source: AMB Express. 2019 Dec 3;9:194. doi: 10.1186/s13568-019-0915-1 (PMC6890922; doi:10.1186/s13568-019-0915-1)
Supplement: Supplementary file 1 — Additional file 1: Table S1. Primers for real-time quantitative PCR. Table S2. Systems for real-time quantitative PCR. [file 13568_2019_915_MOESM1_ESM.docx]

**Additional file.**

“Anti-HIV, Antitumor and Immunomodulatory Activities of Paclitaxel from Fermentation Broth Using Molecular Imprinting Technique”

“Anti-HIV, Antitumor and Immunomodulatory Activities of Paclitaxel from Fermentation Broth Using Molecular Imprinting Technique”

Junhyok Ryang ^a 1^, Yan Yan ^a 1^, Yangyang Song, Fang Liu ^a^ ^*^and Tzi Bun Ng^b*^

^a^ Department of Microbiology, The Key Laboratory of Molecular Microbiology and Technology, Ministry of Education, Nankai University, Tianjin, 300071, China

^b^School of Biomedical Sciences, Faculty of Medicine, The Chinese University of Hong Kong, Shatin, New Territories, Hong Kong, China

*Corresponding author: liufang312@nankai.edu.cn, Phone +86 22-23509491, Fax 52+(444) 8262372

[b021770@mailserv.cuhk.edu.hk](mailto:b021770@mailserv.cuhk.edu.hk), Phone+852 2096872, Fax +852 26035123

Table S1 Primers for real-time quantitative PCR

| Gene | Primers | | Products |
| --- | --- | --- | --- |
| IL-2 | upstream: | 5’-TGAACTTGGACCTCTGCG-3’ | 220bp |
|  | downstream: | 5’-AGGGCTTGTTGAGATGATGC-3’ |  |
| IL-4 | upstream: | 5’-TCCTGCTCTTCTTTCTCG-3’ | 537bp |
|  | downstream: | 5’-ATGCTCTTTAGGCTTTCC-3’ |  |
| IL-6 | upstream: | 5’-TTCTTGGGACTGATGCTG-3’ | 380bp |
|  | downstream: | 5’-CTGGCTTTGTCTTTCTTGTT-3’ |  |
| IL-10 | upstream: | 5’-ACCAAAGCCACAAAGCAG-3’ | 249bp |
|  | downstream: | 5’-GGAGTCGGTTAGCAGTATG-3’ |  |
| IFN-γ | upstream: | 5’-TGAGACAATGAACGCTAC-3’ | 142bp |
|  | downstream: | 5’-TTCCACATCTATGCCACT-3’ |  |
| TNF-α | upstream: | 5’-CTGTGAAGGGAATGGGTGTT-3’ | 384bp |
|  | downstream: | 5’-CAGGGAAGAATCTGGAAAGGTC-3’ |  |
| GAPDH | upstream: | 5’-TCAACGGCACAGTCAAGG-3’ | 470bp |
|  | downstream: | 5’-ACCAGTGGATGCAGGGAT-3’ |  |

Table S2 Systems for real-time quantitative PCR

| composition | volume |
| --- | --- |
| cDNA | 2.0 μL |
| Up primer | 0.8 μL |
| Down primer | 0.8 μL |
| ROX Reference Dye | 0.4 μL |
| SYBR Premix Ex Taq Ⅱ(2×) | 10.0 μL |
| ddH2O | 6.0 μL |
